# Dihydromikanolide Inhibits ROS‐Mediated NLRP3 Inflammation via Antioxidant Nrf2 Activation and Mitophagy Induction in LPS/ATP‐Stimulated Macrophages

**DOI:** 10.1111/jcmm.70803

**Published:** 2025-08-26

**Authors:** You‐Cheng Hseu, Yu‐Fang Tseng, Jhih Ke‐Hseu, Sudhir Pandey, Siang‐Jyun Chen, Kai‐Yuan Lin, Hsueh‐Wei Chang, Tzong‐Der Way, Chuan‐Chen Lee, Jhih‐Hsuan Hseu, Hsin‐Ling Yang

**Affiliations:** ^1^ Department of Cosmeceutics, College of Pharmacy China Medical University Taichung Taiwan; ^2^ Department of Health and Nutrition Biotechnology Asia University Taichung Taiwan; ^3^ Chinese Medicine Research Center China Medical University Taichung Taiwan; ^4^ Research Center of Chinese Herbal Medicine China Medical University Taichung Taiwan; ^5^ Institute of Nutrition, College of Health Care China Medical University Taichung Taiwan; ^6^ Department of Life Sciences National Taiwan Normal University Taipei Taiwan; ^7^ Department of Medical Research Chi Mei Medical Center Tainan Taiwan; ^8^ Department of Biotechnology Chia Nan University of Pharmacy and Science Tainan Taiwan; ^9^ Department of Biomedical Science and Environmental Biology Kaohsiung Medical University Kaohsiung Taiwan; ^10^ Department of Life Sciences China Medical University Taichung Taiwan; ^11^ Department of Dermatology Chang Gung Memorial Hospital and Chang Gung University College of Medicine Kaohsiung Taiwan

**Keywords:** dihydromikanolide, macrophages, mitophagy, NLRP3, Nrf2

## Abstract

Dihydromikanolide (DHK) is a natural product in *Mikania* species. We examined the anti‐inflammatory molecular mechanisms of DHK employing in vitro RAW264.7 macrophages and in vivo BALB/c mice under LPS/ATP stimulation. We found that DHK suppressed NLRP3 inflammasome, procaspase‐1 activation and then pro‐inflammatory IL1β expression in LPS/ATP‐stimulated RAW264.7 cells. Notably, DHK‐triggered autophagy in RAW264.7 cells was demonstrated by increased LC3‐II accumulation, p62/SQSTM1 expression, Beclin‐1/Bcl‐2 ratio and PI3K/AKT/mTOR phosphorylation. Besides, DHK increased Parkin and Pink‐1 protein expressions implying mitophagy induction in RAW264.7 cells. Interestingly, DHK enhanced Nrf2 nuclear translocation and provoked antioxidant HO‐1, NQO‐1 and γ‐GCLC expressions in RAW264.7 cells. Nrf2 knockdown reversed DHK‐inhibited LPS/ATP‐stimulated IL1β expression in RAW264.7 cells. Interestingly, LPS/ATP‐stimulated NLRP3 inflammasome and IL1β expression were inhibited by DHK, Mito‐TEMPO (a mitochondrial ROS inhibitor), or *N*‐acetylcysteine (a ROS inhibitor). In vivo study revealed that DHK attenuated wet/dry weight ratio of lung tissue, lung neutrophil intrusions and pulmonary oedema, and reduced the increased total cells, neutrophils, TNFα and IL1β expression in bronchoalveolar lavage fluid (BALF) in LPS‐stimulated BALB/c mice. DHK alleviated LPS‐induced pathological alterations of lung through inhibiting NLRP3 inflammation, enhancing antioxidant Nrf2 pathway and inducing mitophagy in LPS‐stimulated BALB/c mice. Dihydromikanolide may be a potential therapeutic agent for inflammatory diseases.

## 1 Introduction

Immune cells such as macrophages initiate the inflammatory processes by identifying the danger signals when there is a tissue injury [1]. During inflammation, lipopolysaccharide (LPS) can stimulate numerous signals within macrophages [2] and then enhance the activation of redox‐sensitive transcription factors such as nuclear factor κB (NFκB) [3, 4]. These activated transcription factors subsequently induce their intermediary genes, inducible NO synthase (iNOS) and cyclooxygenase‐2 (COX‐2) and therefore trigger diverse pro‐inflammatory factors comprising prostaglandin E_2_ (PGE_2_) and nitric oxide (NO), which are responsible for the progression and development of inflammatory diseases [4, 5]. LPS‐activated macrophages in immune cells can lead to reactive oxygen species (ROS) generation that drives oxidative stress‐instigated nod‐like receptor pyrin domain‐containing 3 (NLRP3) inflammation [6]. Notably, the stressed and dying cells frequently release ATP, which is sensed as a damage‐associated molecular pattern to trigger NLRP3 inflammation [7]. The NLRP3 is a frequently investigated inflammasome protein complex. By exploiting the complex signalling cascades of the NLRP3 inflammasome, a range of approaches can be applied for its repression including inhibiting NLRP3 inflammasome and caspase‐1 activation, and neutralising inflammatory cytokines like IL1β secreted by the NLRP3 inflammasome, which are linked to various cancers and age‐related diseases [8, 9].

Recent research has elucidated that autophagy plays a substantial role in both the aetiology and progression of inflammation [10]. Elimination of the NLRP3 inflammasome and cytokines by autophagy in macrophages can inhibit the inflammasome instigation and inflammatory response [11]. Autophagosomes integrate with lysosomes to produce autolysosomes, which degrade engulfed components by lysosomal hydrolases in the process of autophagy. The microtubule‐associated protein light chain 3 (LC3) is an important soluble autophagic protein widely distributed in mammalian cells. LC3 exists in membrane‐bound LC3‐II form (transformed from LC3‐I) and in the cytoplasm as LC3‐I (non‐lipidated); however, the conversion of LC3‐I to LC3‐II is essential for the autophagy process. p62 (also known as SQSTM1) is a key scaffold protein that modulates the autophagic flux by binding with both ubiquitinated cellular waste cargo and LC3, resulting in the clearance of damaged proteins/organelles, and it undergoes self‐degradation during autophagy [12]. Cells eliminate non‐functional mitochondria by a precise mechanism of autophagy known as mitophagy. There is evidence that Parkin and PTEN‐induced kinase 1 (Pink‐1) are vital for mitochondrial homeostasis and mitophagy. Parkin mediates the mitophagy quality control pathway since it only attaches to damaged mitochondria, and mitophagy is hindered when Parkin‐Pink‐1 signalling is disrupted [13]. The instigation of the NLRP3 inflammasome can be subdued by eradicating the NLRP3 inflammasome through mitophagy induction in macrophages [11]. The accumulation of malfunctioning depolarized mitochondria occurs due to an impairment of the autophagy process, which releases abnormal levels of inflammasome activators such as reactive oxygen species (ROS) or mitochondrial DNA (mtDNA) [14]. Numerous investigations have confirmed that autophagy irregularities ensuing from obliteration of ATG16, BECLIN1 or LC3B cause an accretion of damaged mitochondria and mitochondrial ROS, which in turn triggers inflammation allied to NLRP3 inflammasome activation and elevated IL‐1β and IL‐18 levels. However, by digesting dysfunctional mitochondria, mitophagy thwarts the release of ROS from the mitochondria, preventing inflammasome activation and limiting the synthesis of inflammatory cytokines [14, 15].

ROS generation by external stimuli such as LPS/ATP promotes NLRP3 inflammasome response in animal tissues and cultured macrophages. There is a crosstalk between the transcription factor NF‐E2‐related factor 2 (Nrf2) and ROS‐mediated NFκB/NLRP3 inflammasome pathways [16]. Although cells continuously manufacture Nrf2, there is little transit of this protein to the nucleus under normal circumstances because it is degraded by binding to Kelch‐like ECH‐associated protein 1 (KEAP1), through the 26S proteasome. Nevertheless, when KEAP1 is exposed to electrophiles, it becomes alkylated or oxidised (inactivated) and loses its capacity to degrade Nrf2. Additionally, autophagy receptor p62‐mediated KEAP1 sequestration and degradation also contribute to Nrf2 activation during elevated ROS levels [17].

Nrf2 facilitates cellular antioxidant defences by governing the expression of genes that encode antioxidant/detoxifying enzymes such as hemeoxygenase‐1 (HO‐1), γ‐glutamate‐cysteine ligase catalytic subunit (γ‐GCLC) and NAD(P)H: quinone acceptor oxidoreductase‐1 (NQO‐1) [18]. An essential enzyme in heme breakdown, HO‐1 generates iron, biliverdin and carbon monoxide–all of which have anti‐inflammatory and antioxidant properties. NQO‐1 is a key enzyme in the detoxification of quinones and other electrophiles. γ‐glutamylcysteine, a precursor for glutathione (GSH) production, is produced by the ATP‐dependent ligation of L‐glutamate and L‐cysteine by GCLC. The tripeptide GSH is crucial for preserving redox homeostasis and shielding cells from oxidative damage [17].

Evidence suggests that ROS‐mediated NFκB and NLRP3 inflammasome are inhibited by antioxidant Nrf2 activating compounds [16]. According to the literature, dihydromikanolide (DHK) belongs to a class of natural sesquiterpene lactones reported from several *Mikania* species [19, 20]. Ethnobotanically, *Mikania* extracts have been widely used in traditional medicine as an analgesic, anti‐rheumatic, anti‐asthmatic, vermifuge, febrifuge and anti‐inflammatory agent [19, 21]. Further, *Mikania micrantha* (aqueous extracts) has demonstrated encouraging outcomes in causing human leukaemia K562 and cervical Hela cancer cells to undergo apoptosis [22]. Additionally, DHK exhibited cytotoxic activities on glioblastoma (U251), breast (MCF‐7) and lung (SKLU‐1) cancer cells [23]. Mechanistically, DHKs along with other sesquiterpene lactones derived from the *Mikania* genus were shown to be DNA polymerase inhibitors [19]. In addition to anti‐cancer activities, DHK was shown to inhibit pathological inflammation in 12‐O‐Tetradecanoylphorbol Acetate (TPA)‐induced edema in a mouse ear model [23]; nevertheless, the molecular mechanisms were yet not known. In the current study, we evaluated the molecular mechanisms of DHK against LPS/ATP‐stimulated ROS‐mediated NFκB and NLRP3 inflammasome in murine RAW264.7 macrophages via antioxidant Nrf2 pathways and mitophagy induction. RAW264.7 cells are a widely accepted cellular model to investigate the LPS‐stimulated inflammatory responses. They exhibit most of the inflammasome‐associated protein complexes that play a pivotal role during pyroptosis and inflammatory processes, and therefore, serve as a useful cellular model to investigate inflammatory phenomena [24, 25]. Moreover, in our laboratory, RAW264.7 cells are well‐established cellular models to study inflammation‐associated cellular mechanisms [26]. The in vivo anti‐inflammation activity of DHK was further interrogated in LPS‐stimulated BALB/c mice.

## 2 Materials and Methods

### 2.1 Chemicals and Reagents

The cell culture reagents, including Dulbecco's Modified Eagle's medium (DMEM), fetal bovine serum (FBS), glutamine and antibiotic solution penicillin–streptomycin, were obtained from the Invitrogen/GIBCO BRL (Grand Island, NY, USA). Dihydromikanolide (DHK; purity > 97%) was purchased from ALFA Biotech (Chengdu, China). LPS (from *Escherichia coli* 055: B5), 3‐(4,5‐dimethylthiazol‐2‐yl)‐2,5‐diphenyltetrazolium bromide (MTT), 2′,7′‐dihydrofluorescein‐diacetate (DCFH_2_‐DA) and *N*‐acetylcysteine (NAC) were acquired from Sigma‐Aldrich (St. Louis, MO, USA). Calbiochem (La Jolla, CA, USA) supplied cyclosporin A and 4′, 6‐Diamidino‐2‐phenylindole dihydrochloride (DAPI), while Invitrogen/GIBCO BRL (Grand Island, NY, USA) provided ATP and Mito‐TEMPO. NLRP3 antibody was obtained from Biorbyt (Cambridge, UK). IL1β, HO‐1 and Parkin antibodies were purchased from Abcam (Cambridge, UK). Antibodies against Pink‐1 and γ‐GCLC were supplied by Genetic Technology Inc. (Miami, FL, USA). β‐actin, Nrf2, p70S6 kinase, caspase‐1, GAPDH, NQO‐1 and COX‐2 antibodies were procured from Santa Cruz (Heidelberg, Germany). Cell Signalling Technology Inc. (Danvers, MA) provided antibodies against Bax, AKT, p‐AKT, p62, Beclin‐1, PI3K, p‐PI3K, p‐mTOR, mTOR, LC3B, p38, p‐p38, iNOS and histone H3. The other chemicals were obtained from either Merck (Darmstadt, Germany) or Sigma (St. Louis, MO, USA).

### 2.2 Cell Culture Maintenance and Treatment

Murine derived RAW264.7 macrophage cells were attained from American Type Culture Collection (ATCC, Rockville, MD, USA) and maintained in DMEM comprising glutamine (2 mM), penicillin–streptomycin (1%) and FBS (10%) in a humidified incubator at 37°C supplied with 5% CO_2_ [26]. The cultured cells were subjected to DHK for 1 h incubation and then rinsed with phosphate buffer saline (PBS). Next, the fresh culture medium containing or lacking LPS (1 μg/mL for 5.5 h) and ATP (5 mM for 0.5 h) was added to the cells.

### 2.3 MTT Assay

In a 24‐well plate, 1 × 10^5^ RAW264.7 macrophages/well were grown. Afterwards, they were subjected to 0–20 μM DHK for 24 h incubation. After DHK treatment, the MTT reagent (400 μL of 0.5 mg/mL) was added to the cells in culture medium for 1 h. Post incubation, the formazan crystals produced were solubilised in dimethyl sulfoxide (DMSO, 400 μL). The cell viability was assessed by determining the absorbance employing a microplate reader at 570 nm (BioTek Instruments, Winooski, VT, USA) [27] and expressed as the percentage viability of cells by comparison with vehicle‐treated controls (as 100%).

### 2.4 ROS Determination

The ROS release was determined using DCFH_2_‐DA reagent (Molecular Probes, Invitrogen), as described previously [28]. 1 × 10^5^ RAW264.7 cells/well were pre‐treated in a 24‐well plate with DHK (0 or 12.5 μM) for 1 h in the presence or lack of LPS (1 μg/mL) for 5.5 h, subsequently by ATP (5 mM) for 0.5 h. Next, the cells were incubated with 10 μM DCFH_2_‐DA in culture medium and incubated for 30 min at 37°C. Finally, PBS wash was given to the cells and ROS levels indicated by fluorescence intensity of DCF was detected using a fluorescence microscope (λ_ex_/λ_em_: 485/530 nm) (Olympus, Center Valley, PA, USA).

### 2.5 Western Blotting

1 × 10^6^ RAW264.7 cells/dish were grown in a 6 cm culture dish and subjected to DHK (0–12.5 μM) treatment with or without LPS (1 μg/mL for 5.5 h) and ATP (5 mM for 0.5 h) for the specified durations. Subsequently, the cells were trypsinized, collected and bathed in ice‐cold PBS. Afterward, the total, cytosolic and nuclear protein fractions were isolated using commercially available protein purification reagents (Pierce Biotechnology, Rockford, IL, USA). The same amount (50 μg) of denatured protein samples were electrophoretically separated using 8%–15% sodium dodecyl sulfate‐polyacrylamide gel (SDS‐PAGE) and then transferred onto a polyvinylidene fluoride (PVDF) membrane. Then, 5% non‐fat dry milk powder was used to block the non‐specific attachment of proteins on membranes for 30 min at room temperature. After blocking, the membranes were treated with primary antibody overnight at 4°C, rinsed with phosphate‐buffered saline containing 0.1% Tween 20 detergent (PBST), and then incubated for another 2 h at room temperature with goat anti‐rabbit or anti‐mouse secondary antibody conjugated with horseradish peroxidase [29]. Using a commercially available program (AlphaEase, Genetic Technology Inc. Miami, FL, USA), the densitometric analysis of protein band intensities was performed with control represented as 1‐fold or 100%.

### 2.6 Immunofluorescence Staining

1 × 10^4^ RAW264.7cells/well were grown on an eight well chamber slide and treated for 1 h with DHK (0 or 12.5 μM) and next, incited for 5.5 h with LPS (1 μg/mL) followed by ATP (5 mM) for 0.5 h. The immunostaining was performed as described previously [2]. Following treatments, the cells were fixed for 15 min using 2% paraformaldehyde, permeabilised for 10 min using 0.1% Triton X‐100, washed with PBS and then blocked using 10% FBS in PBS. After 2 h of incubation with primary antibodies in 1.5% FBS, the cells were treated for 1 h in 6% bovine serum albumin (BSA) with a secondary antibody conjugated to fluorescein isothiocyanate (FITC, 488 nm). Cells were counterstained with DAPI, rinsed with PBS and detected under a confocal microscope (630 × magnification) (Leica TCS SP2, Heidelberg, Germany).

### 2.7 siRNA Transfection

The transient siRNA transfections in RAW264.7 macrophage cells were performed using Lipofectamine RNAiMAX reagent (Invitrogen, Grand Island, NY, USA). The RAW264.7 cells were seeded (4 × 10^5^ cells) in a 6‐well culture plate with DMEM medium containing 10% FBS and allowed to grow till 60% confluence. The transfection was done as described previously [30]. 250 μL of Opti‐MEM and 5 μL of RNAiMAX were combined, and they were incubated for 5 min at room temperature. A second tube containing 250 μL of Opti‐MEM was used to dilute siRNAs (100 pM). Both the tubes were mixed to create a transfection complex (siRNA/RNAiMAX) of 500 μL volume and incubated for a further 25 min at room temperature. The resulting transfection complex (500 μL) was then added to the cells in the 6‐well plate, making a volume of 1 mL with opti‐MEM. 6 h after transfection, 2 mL of regular culture media were added, and cultured at 37°C. Next, the cells were treated with DHK (0 or 12.5 μM) for 1 h followed by LPS (1 μg/mL) for 5.5 h and ATP (5 mM) for 0.5 h.

### 2.8 Establishment of the Acute Lung Injury Mouse Model

Six weeks of female BALB/c mice (20–24 g) were acquired from the National Laboratory Animal Center (Taipei, Taiwan) and caged in an animal facility at China Medical University (CMU, Taichung) in specialised pathogen‐free environments and had unrestricted access to food and water. The mice were acclimatised in a 12‐h cycle of light and dark. The CMU Animal Ethics Research Board and Institutional Animal Care Committee (IACUC 2018–065) authorised standard protocols for all animal experiments. The mice were arbitrarily segregated into five groups (*n* = 6): (1) control, (2) DHK (6 mg/kg), (3) LPS (1 mg/kg), (4) LPS + DHK (3 mg/kg) and (5) LPS + DHK (6 mg/kg). Mice were administered DHK (3 and 6 mg/kg) by intraperitoneal injection. After 4 h, mice were anaesthetised via an intraperitoneal injection with Zoletil 50 (100 mg/kg); 1 mg/kg LPS was infused into the trachea of mice to establish an acute lung injury model through non‐exposed tracheal instillation for 24 h. The same volume of physiological saline was injected into the control mice. The mice were sacrificed at 24 h after LPS stimulation; lung tissue and bronchoalveolar lavage fluid (BALF) were collected.

### 2.9 Bronchoalveolar Lavage Fluid (BALF) Analysis

The mice were euthanised, and trachea was exposed. To acquire BALF, 0.7 mL of PBS was injected into the trachea and aspirated the fluid thrice [31, 32]. Briefly, the BALF samples were spun at 3000 rpm for 10 min at 4°C. After that, the sedimented cells were resuspended in PBS, and a hemocytometer was used to enumerate the total number of cells. Cells from BALF (200 μL) were centrifuged at 600 rpm and spun onto glass slides by a cytospin, and then the cells were stained using Wright‐Giemsa staining. Combinations of methylene blue (basic dyes) and eosin (acidic dyes) that stain red constitute Wright‐Giemsa staining. The basic cell components like haemoglobin and eosinophilic granules stain red or orange, while acidic fractions like the basophilic granules, nucleus and cytoplasmic RNA stain blue or purple.

### 2.10 Enzyme‐Linked Immunosorbent Assay (ELISA) of BALF

In BALF, the amount of TNF‐α and IL1β was estimated using an ELISA technique utilising a commercially available kit (R&D Systems, Minneapolis, MN, USA) following the manufacturer's instructions [31, 32]. Using a microplate reader, the absorbance was measured at 450 nm.

### 2.11 Wet/Dry Weight Ratio of Left Lung

The degree of pulmonary oedema is indicative of the severity of lung inflammation, which is determined by examining the wet/dry weight ratio of lung tissue. The wet weight of the left lung tissue of mice was determined immediately after sacrifice and then lung weight was measured again after drying in an oven at 60°C for 72 h [33].

### 2.12 Immunoblotting Analysis of Right Lung From Experimental Mice

Total proteins were extracted from the right lung by using RIPA buffer, and each sample (50 μg) was used to inspect the expression of NLRP3, procaspase‐3, IL1β, LC3‐I/II, Parkin, and Pink‐1 following a standard immunoblotting procedure as previously described [34].

### 2.13 Statistical Analysis

The statistical significance among all the data was examined using an analysis of variance (ANOVA) method with Dunnett's pair‐wise comparison. The results were expressed as mean ± SD (*n* = 3–6). *p < 0.05; **p < 0.01; ***p < 0.001 when compared with control cells; ^#^ *p* < 0.05; ^##^ *p* < 0.01; ^###^ *p* < 0.001 when compared with 3‐MA‐, LPS/ATP‐, or LPS‐stimulated cells.

## 3 Results

### 3.1 The Effect of Dihydromikanolide (DHK) on Cell Viability of RAW264.7 Macrophages

**FIGURE 1:**
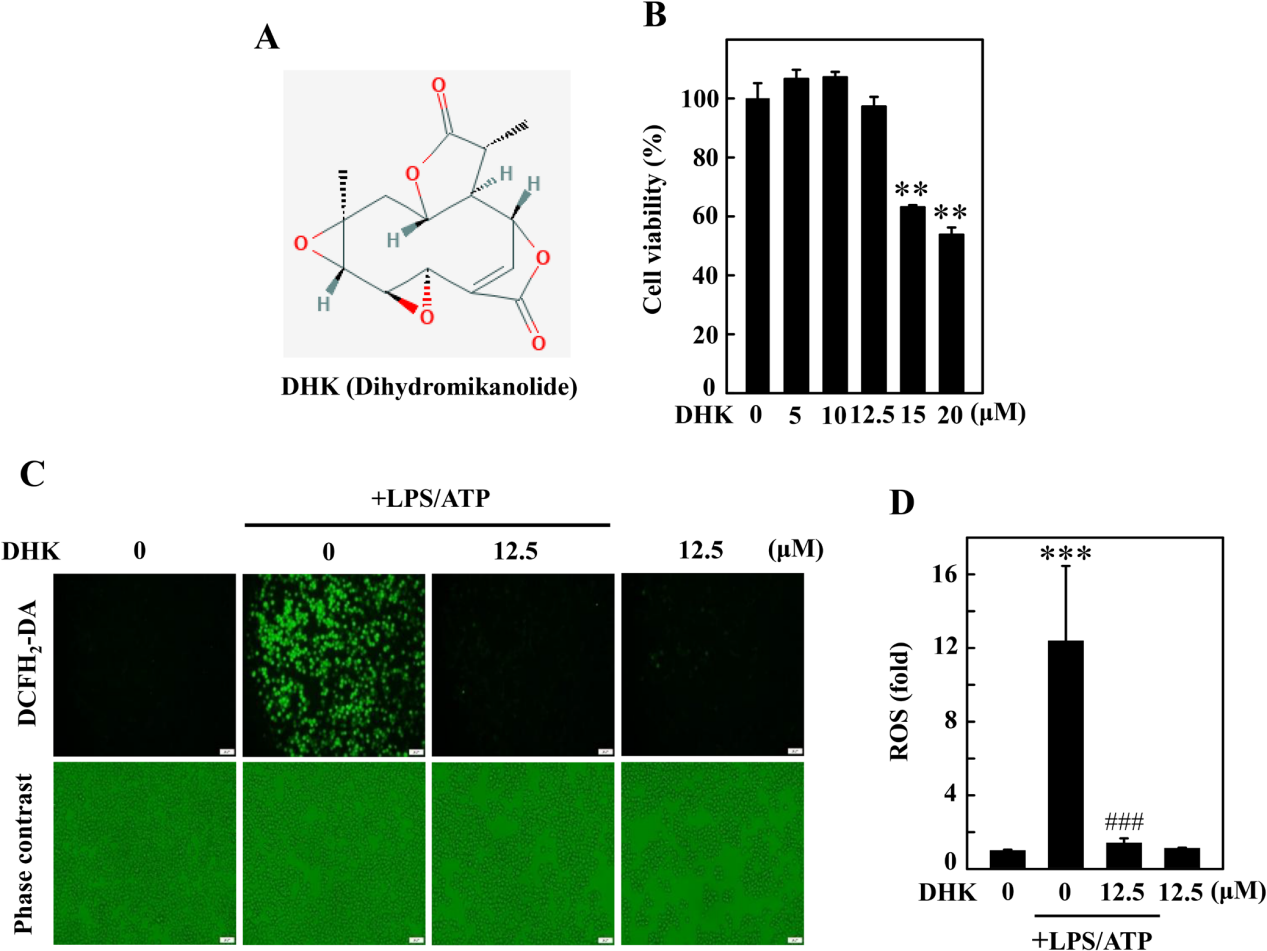
Dihydromikanolide (DHK) attenuated ROS generation in LPS/ATP‐stimulated RAW 264.7 macrophages. (A) Structure of DHK. (B) The cell viability was determined by MTT assay. Cells were treated with DHK (0–20 μM) for 24 h. (C, D) Cells were pre‐treated with DHK (0–12.5 μM for 1 h) and then stimulated with LPS (1 μg/mL for 5.5 h) following by ATP (5 mM for 0.5 h). The level of intracellular ROS was measured by DCF fluorescence using fluorescence microscopy.

To examine the anti‐inflammatory properties of DHK (Figure 1A), the cytotoxic effects of various concentrations of DHK ranging from 0 to 20 μM for 24 h on RAW264.7 macrophages were investigated. The MTT analysis exhibited that there were no obvious effects on the viability of RAW264.7 cells up to the 12.5 μM concentration, but a substantial decrease was detected with 15 and 20 μM treatment (Figure 1B). Therefore, the non‐cytotoxic doses of DHK, that is, ≤ 12.5 μM, were selected for in vitro studies and to investigate their effects on LPS/ATP‐incited NFκB and NLRP3 inflammation in RAW264.7 cells.

### 3.2 DHK Diminishes LPS/ATP‐Incited ROS Release in RAW264.7 Cells

The accretion of LPS/ATP‐instigated ROS in macrophages can intensify the inflammatory outcomes [35]. When RAW264.7 cells were instigated with LPS/ATP for 6 h (LPS [1 μg/mL] for 5.5 h and then ATP [5 mM] for 0.5 h), then it prompted the intracellular ROS release as shown in Figure 1C,D. However, pretreatment with DHK (12.5 μM for 1 h) in LPS/ATP‐induced macrophages remarkably attenuated ROS generation (Figure 1C,D).

### 3.3 DHK Inhibits NFκB Transcriptional Activation in LPS/ATP‐Instigated RAW264.7 Cells

**FIGURE 2:**
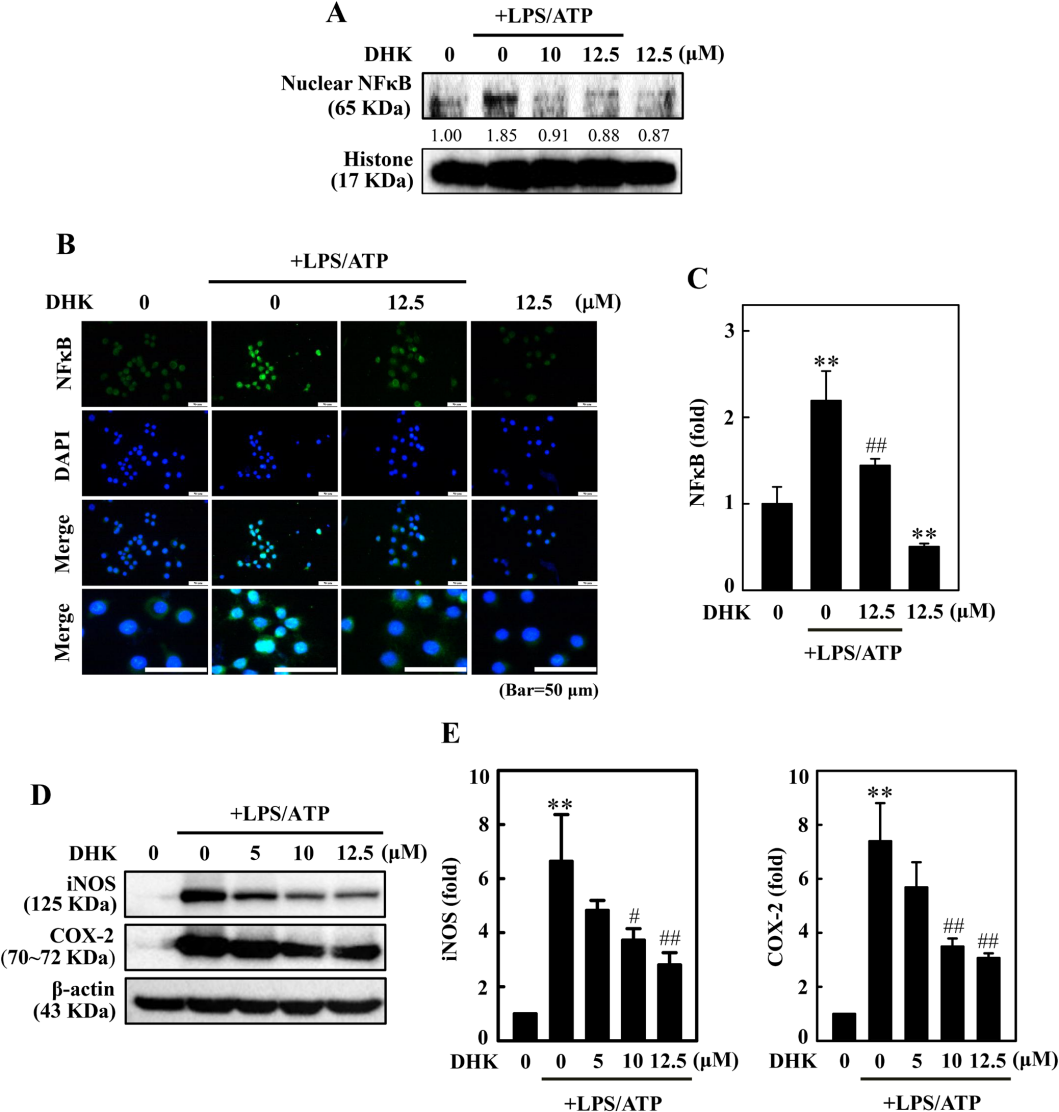
DHK suppressed NFκB signalling pathway in LPS/ATP‐stimulated RAW264.7 cells. Cells were treated with DHK (0–12.5 μM) for 1 h, and then LPS (1 μg/mL) for 5.5 h followed by ATP (5 mM) for 0.5 h. (A) The nuclear NFκB expression was determined by western blotting. (B, C) NFκB expression was measured using immunofluorescence staining. Cells were incubated with anti‐NFκB antibody followed by secondary antibody labelled with FITC. A confocal microscope was used to visualise the subcellular localization of NFκB. (D, E) Inflammatory iNOS and COX‐2 expressions were determined using western blotting. β‐actin served as internal control.

In vitro studies employing LPS‐primed macrophages have shown the activation of NFκB and NLRP3 inflammasome, and ATP drives the cleavage of caspase‐1 [14]. NFκB expression was measured by Western blotting and immunofluorescence. Here we examined whether pretreatment with DHK (0–12.5 μM for 1 h) could suppress the LPS/ATP (for 6 h)‐induced NFκB (p65, the subunit of NFκB) expression in RAW274.7 cells. The results exhibited that a huge increase of NFκB (p65) expression upon LPS/ATP instigation was markedly inhibited by DHK pretreatment in a concentration‐dependent manner (Figure 2A). Immunofluorescence was performed to examine NFκB (p65) nuclear translocation. We found that NFκB (p65) was cytosolic in un‐stimulated RAW264.7 cells, but nuclear NFκB (p65) was significantly enriched upon LPS/ATP stimulation (Figure 2B,C). However, pretreatment with DHK for 1 h suppressed LPS/ATP‐stimulated nuclear translocation of NFκB (p65) in RAW264.7 cells.

### 3.4 DHK Inhibited LPS/ATP‐Stimulated iNOS and COX‐2 Expression in RAW264.7 Cells

To examine the anti‐inflammatory activities of DHK, RAW264.7 cells were treated with DHK (0–12.5 μM) for 1 h and later incited for 5.5 h with LPS (1 μg/mL) and then for 0.5 h with ATP (5 mM). The outcomes showed that LPS/ATP stimulation alone markedly enhanced iNOS and COX‐2 expression; but treatment with DHK remarkably decreased iNOS and COX‐2 expression in a dose‐dependent manner (Figure 2D,E).

### 3.5 DHK Suppressed LPS/ATP‐Incited IL1β Expression Through NLRP3 Inflammasome and Procaspase‐1 Inhibition in RAW264.7 Cells

**FIGURE 3:**
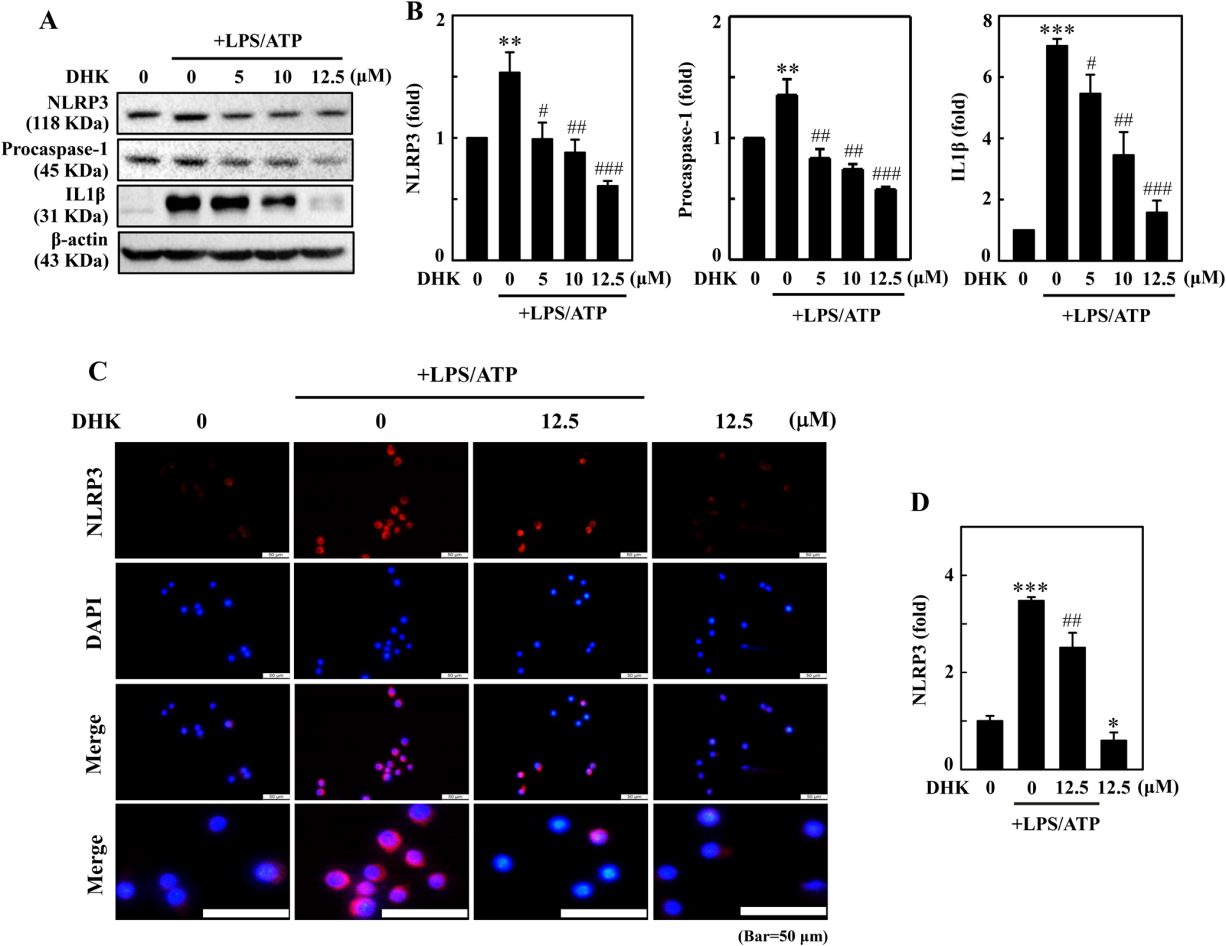
DHK suppressed NFκB and NLRP3 inflammasome in LPS/ATP‐stimulated RAW264.7 cells. Cells were treated with DHK (0–12.5 μM) for 1 h, and then LPS (1 μg/mL) for 5.5 h followed by ATP (5 mM) for 0.5 h. (A, B) The NLRP3, procaspase‐1, and IL1β expressions were determined by western blotting. (C, D) NLRP3 expression was measured using immunofluorescence staining.

Here, we inspected whether DHK (0–12.5 μM) could curb the LPS/ATP‐incited NLRP3 and procaspase‐1 initiation in RAW264.7 cells. As displayed by western blotting, the NLRP3 inflammasome was prominently amplified in macrophages instigated with LPS/ATP, validating that procaspase‐1 was activated (Figure 3A,B). DHK treatment dose‐dependently repressed NLRP3 and procaspase‐1 expression (Figure 3A,B). Further, owing to LPS/ATP stimulation, there was a notable enhancement in the expression of IL1β which was substantially attenuated by DHK pretreatment (Figure 3A,B). Immunofluorescence staining confirmed that DHK remarkably inhibited LPS/ATP‐stimulated nuclear translocation of NLRP3 in RAW264.7 cells (Figure 3C,D). Our results are also substantiated by previous research findings, which showed that cytoplasmic enrichment of NLRP3 promotes inflammasome assembly, while nuclear localization might enhance the transcriptional activity of the inflammasome [36, 37].

### 3.6 DHK Enhanced Autophagy Through LC3‐II Accumulation and p62 Expression in LPS/ATP‐Stimulated RAW264.7 Cells

**FIGURE 4:**
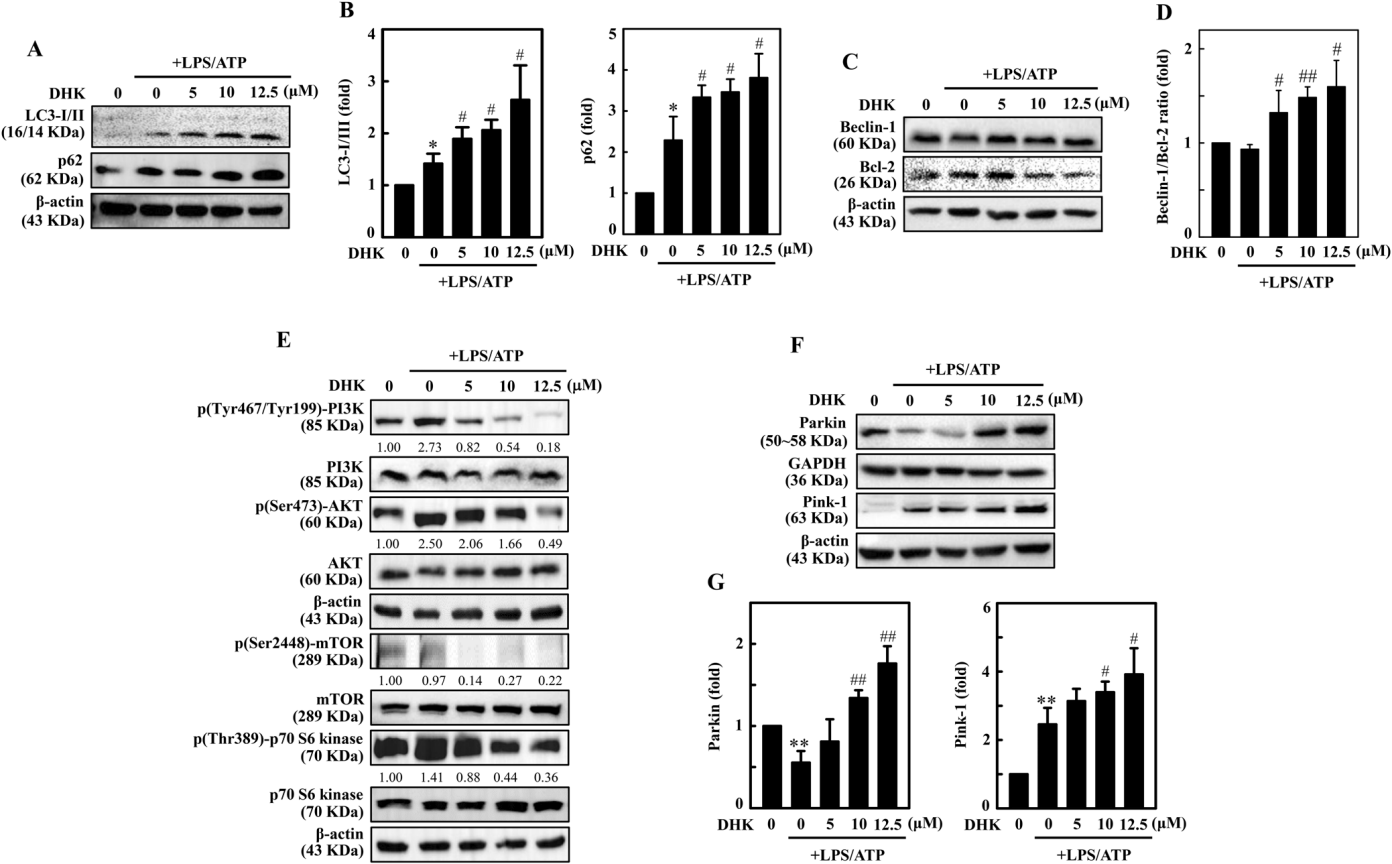
DHK induced autophagy in LPS/ATP‐stimulated RAW264.7 cells. Cells were treated with DHK (0–12.5 μM) for 1 h, and then LPS (1 μg/mL) for 5.5 h followed by ATP (5 mM) for 0.5 h. (A, B) LC3‐I/LC3‐II and p62 expression was estimated by western blotting. (C, D) Beclin‐1 and Bcl‐2 expression shown by western blot analysis. The relative changes of Beclin‐1/Bcl‐2 ratio was presented in adjacent graph. (E) Dose‐dependent expression of total and phosphorylated forms of PI3K, AKT, mTOR and p70S6 kinase was determined by Western blotting. (F, G) DHK induces mitophagy in LPS/ATP‐stimulated RAW264.7 cells. Parkin and Pink‐1 expression was determined by western blotting.

In inflammation, autophagy plays an important role by affecting homeostasis of inflammatory macrophages. Our results from Western blot analysis displayed that with cumulative concentrations of DHK (0–12.5 μM), LC3‐II accumulation was upregulated in LPS/ATP‐stimulated RAW264.7 cells (Figure 4A,B). The expression of p62 (sequestosome 1, SQSTM1) is used as a marker of autophagy flux which is degraded during autolysosomal clearance [38]. Western blotting analysis demonstrated that DHK increased p62 expression in LPS/ATP‐stimulated RAW264.7 cells (Figure 4A,B). These results implicated that DHK triggered autophagy through LC3‐II accumulation and p62 expression in LPS/ATP‐stimulated RAW264.7 cells.

### 3.7 DHK Increased Beclin‐1 and Bcl‐2 Ratio Contributing to Enhanced Autophagy in LPS/ATP‐Incited RAW264.7 Cells

Beclin‐1 is a key protein in the early autophagosome formation which helps in the recruitment of pre‐autophagosomal proteins, whereas Bcl‐2 binds with Beclin‐1 and antagonises autophagy [39]. The outcome of DHK (0–12.5 μM) on Beclin‐1 and Bcl‐2 association was examined employing Western blotting. The results demonstrated that DHK dose‐dependently increased autophagic Beclin‐1 and decreased anti‐autophagic Bcl‐2 expression in LPS/ATP‐instigated RAW264.7 cells (Figure 4C). Remarkably, DHK increased Beclin‐1 and Bcl‐2 ratio leading to enhanced autophagy (Figure 4D).

### 3.8 DHK Repressed PI3K/AKT/mTOR, and p70S6 Kinase Signalling Pathway Contributing to Enhanced Autophagy in LPS/ATP‐Stimulated RAW264.7 Cells

PI3K/AKT/mTOR signalling pathway involving p70S6 kinase is a crucial regulator of autophagy [40]. Western blotting results showed that in comparison to untreated control, DHK (0–12.5 μM) dose‐dependently downregulated p‐PI3K, p‐AKT, p‐mTOR and p‐p70S6 kinase expressions in LPS/ATP‐stimulated RAW264.7 cells (Figure 4E). Therefore, the findings indicated that DHK triggered autophagy through the suppression of PI3K/AKT/mTOR and p70S6 kinase expression in LPS/ATP‐induced RAW264.7 cells.

### 3.9 DHK Induced Mitophagy in LPS/ATP‐Stimulated RAW264.7 Cells

Damaged mitochondria are removed through a mitochondria‐selective autophagic mechanism, mitophagy, which preserves mitochondrial quality in the presence of stress within cells. Parkin and Pink‐1 proteins play a vital role in mitochondrial quality control and mitophagy [41]. This study showed that LPS/ATP stimulation inhibited Parkin and Pink‐1 expressions in RAW264.7 cells (Figure 4F,G). DHK pre‐treatment induced mitophagy through upregulation of Parkin and Pink‐1 expression, which were recruited to the damaged mitochondria in LPS/ATP‐induced RAW264.7 cells (Figure 4F,G).

### 3.10 DHK Reduced LPS/ATP‐Stimulated NFκB‐ and NLRP3‐Mediated Inflammation Through Enhanced Autophagy in RAW264.7 Cells

**FIGURE 5:**
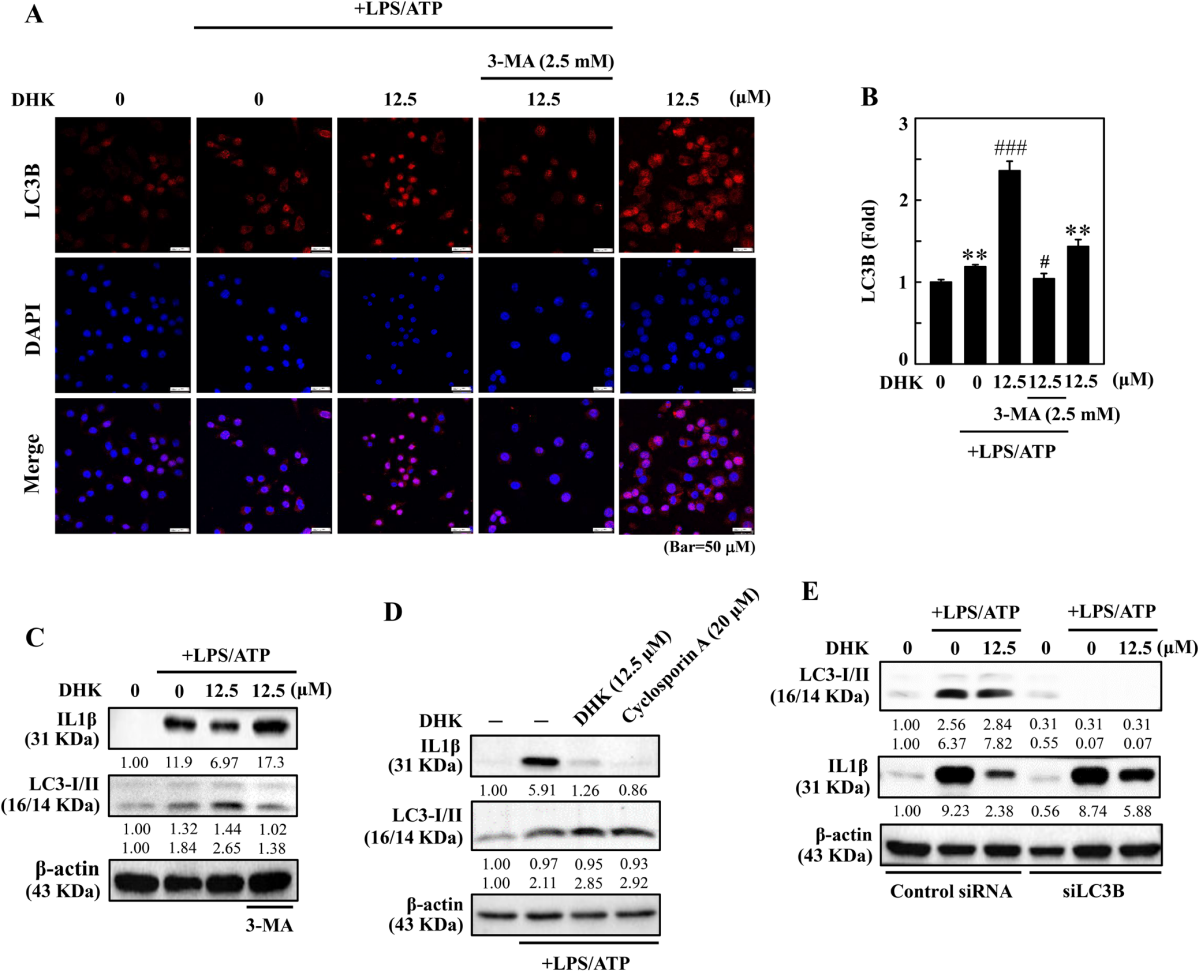
DHK inhibits NFκB and NLRP3 inflammasome activation through autophagy induction in LPS/ATP‐stimulated RAW264.7 cells. (A, B) Cells were treated with DHK (0 and 12.5 μM) without or with 3‐MA (2.5 mM) for 1 h, and then LPS (1 μg/mL) for 5.5 h followed by ATP (5 mM) for 0.5 h. LC3‐I/II expression was determined by immunofluorescence. (C) Cells were treated with DHK (0 and 12.5 μM) without or with 3‐MA (2.5 mM) for 1 h, and then LPS (1 μg/mL) for 5.5 h followed by ATP (5 mM) for 0.5 h. IL1β and LC3‐I/II expression was determined by Western blotting. (D) Cells were treated with DHK (12.5 μM) or NFκB inhibitor cyclosporin A (20 μM) for 1 h, and then LPS (1 μg/mL) for 5.5 h followed by ATP (0.5 mM) for 1 h. IL1β and LC3‐I/II expression was determined by Western blotting. (E) LC3 knockdown attenuated the protective effects of DHK. Cells were first transfected with siRNA that is specific to either LC3B or a non‐silencing control then pre‐treated with DHK (0 and 12.5 μM) for 1 h and then stimulated with LPS (1 μg/mL) for 5.5 h following by ATP (5 mM) for 0.5 h and IL1β or LC3‐I/II expression in both control and siLC3B were determined using western blot analysis.

To interrogate the role of autophagy in the inhibition of NFκB‐ and NLRP3‐mediated inflammatory IL1β expression in LPS/ATP‐incited RAW264.7 cells, the autophagic inhibitor 3‐MA, which blocks early autophagosome formation, was utilised. Immunofluorescence staining revealed that DHK upregulated LC3B expression in LPS/ATP‐stimulated RAW264.7 cells, which were akin to Western blotting results (Figure 5A,B). However, 3‐MA reversed DHK‐enhanced LC3B expression (Figure 5A,B). As shown by Western blotting, DHK inhibited IL1β and enhanced LC3‐II expressions, which were reversed by 3‐MA in LPS/ATP‐stimulated RAW264.7 cells (Figure 5C) and the results showed consistency with immunofluorescence data. In addition, DHK, as well as NFκB inhibitor cyclosporin A (20 μM) treatment suppressed LPS/ATP‐stimulated IL1β expression (Figure 5D), suggesting that DHK inhibited NFκB‐activated IL1β expression in LPS/ATP‐stimulated RAW264.7 cells. Furthermore, the suppression of autophagy through LC3 silencing (siLC3) in RAW264.7 cells reversed DHK (12.5 μM)‐suppressed LPS/ATP‐stimulated IL1β expression (Figure 5E). Thus, the above data suggested that DHK‐triggered autophagy acts as a cellular homeostasis mechanism to suppress the NFκB‐ and NLRP3‐mediated inflammatory IL1β expression.

### 3.11 DHK Upregulated HO‐1, NQO‐1, and γ‐GCLC Expression by Activating Nrf2 Pathway in RAW264.7 Cells

**FIGURE 6:**
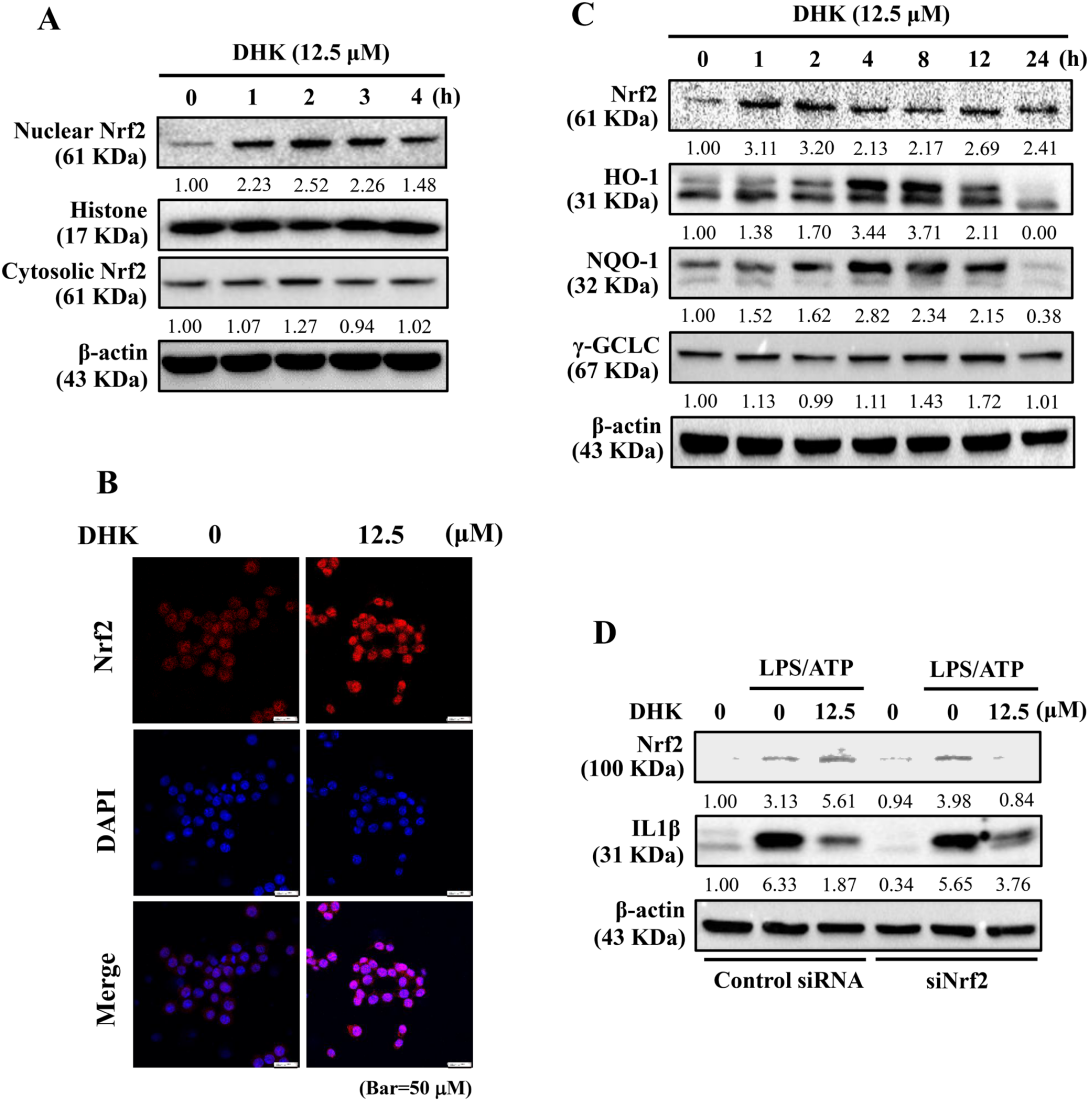
DHK increased Nrf2 signalling pathway leading to antioxidant HO‐1, γ‐GCLC and NQO‐1 expression resulted in inhibiting pro‐inflammatory IL1β expression in RAW264.7 cells. (A) Treatment of cells with DHK (12.5 μM) for 0–4 h to determine Nrf2 levels in the nuclear and cytosolic fractions. (B) Nrf2 expressions were determined by immunofluorescence staining. Cells were treated with DHK (0–12.5 μM) for 2 h. (C) DHK (12.5 μM) for 0–24 h was added to cells, and cell lysates were subjected to an immunoblotting assay to determine the time‐dependent changes in Nrf2, HO‐1, NQO‐1 and γ‐GCLC expression. (D) Nrf2 knockdown attenuated the protective effects of DHK. Cells were transfected with siRNA that is specific to either Nrf2 or a non‐silencing control. Nrf2 or IL1β expression in both control and siNrf2 were examined by western blotting.

Antioxidants HO‐1, NQO‐1 and γ‐GCLC expression considered as an important protective mechanism against cellular oxidative stress is regulated by Nrf2 nuclear translocation and activation [18]. Investigation had found that Nrf2 activation negatively regulates IL1β expression by suppressing ROS‐mediated NLRP3 inflammasome activation [42]. Here, the effects of DHK (12.5 μM for 0–4 h) on nuclear and cytosolic Nrf2 expression was evaluated in RAW264.7 cells using Western blotting and immunofluorescence staining. In the presence of DHK, an enhancement in nuclear Nrf2 translocation was observed with maximum levels at 2 h as demonstrated by Western blotting data (Figure 6A). Consistent with Western blotting data, immunofluorescence staining showed a significant enrichment in Nrf2 nuclear translocation in DHK (12.5 μM for 4 h)‐treated RAW264.7 cells (Figure 6B). Moreover, the immunoblotting results revealed that DHK (12.5 μM for 0–24 h) upregulated Nrf2 and its downstream regulated genes HO‐1, NQO‐1 and γ‐GCLC expression (Figure 6C). Collectively, above data suggested that DHK promoted Nrf2 nuclear translocation and activation, and therefore triggered antioxidant γ‐GCLC, HO‐1 and NQO‐1 expressions in RAW264.7 cells.

### 3.12 Nrf2 Knockdown Reversed DHK‐Inhibited IL1β Expression

The Nrf's crucial contribution in DHK inhibited ROS‐induced NLRP3 inflammasome and IL1β expression in RAW264.7 cells was more ascertained through Nrf2 knockdown examination. In LPS/ATP‐incited RAW264.7 cells, DHK (12.5 μM) caused a conspicuous decline in IL1β expression in control siRNA‐transfected cells, whereas Nrf2 knockdown reversed DHK‐inhibited IL1β expression (Figure 6D). These data suggested that DHK‐triggered antioxidant transcription factor Nrf2 plays a crucial role in the inhibition of LPS/ATP‐incited ROS‐mediated IL1β expression in RAW264.7 cells.

### 3.13 JNK, PI3K/AKT and p38‐Facilitated Nuclear Nrf2 Translocation in DHK‐Treated RAW264.7 Cells

**FIGURE 7:**
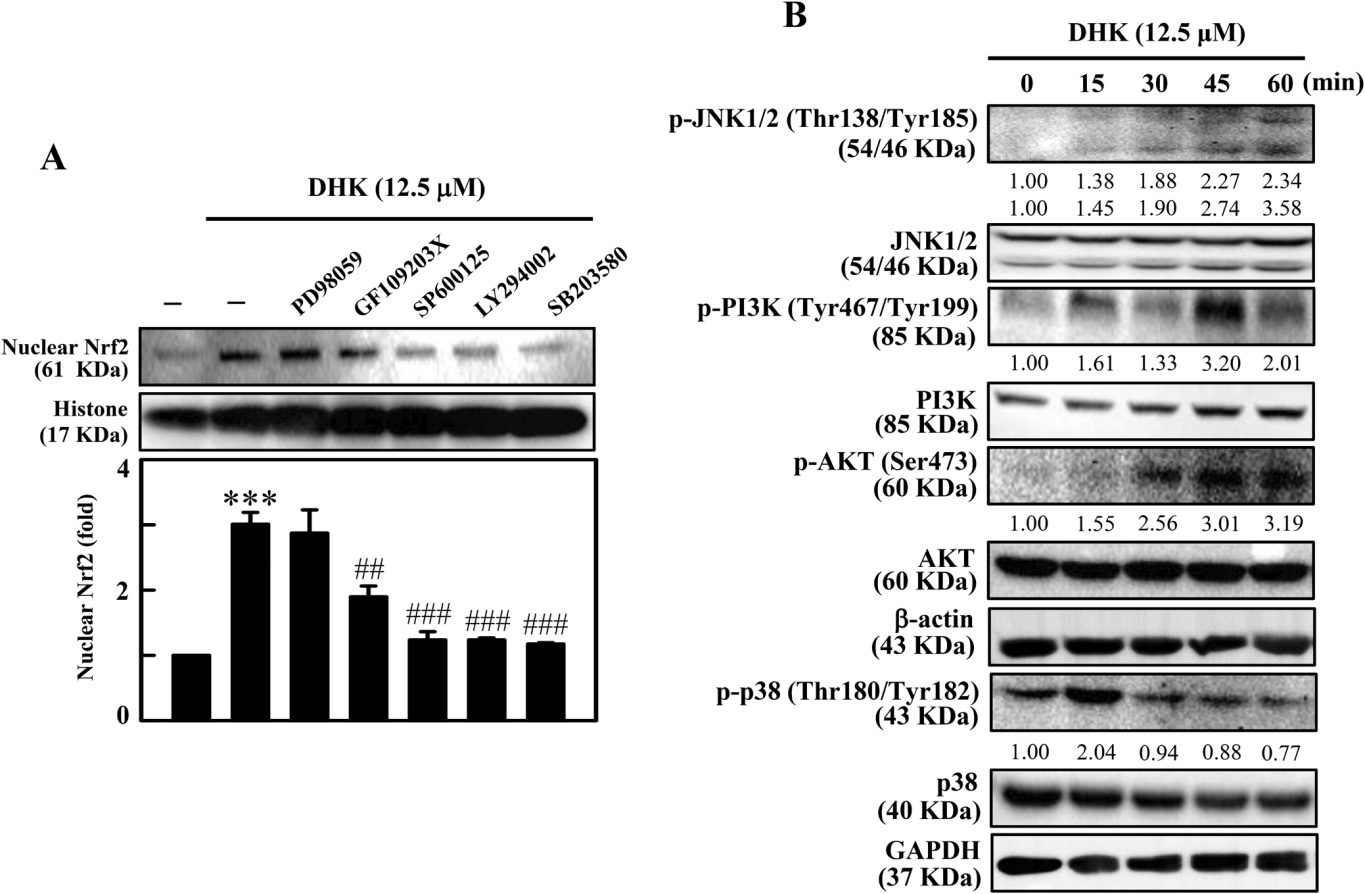
Nrf2 activation by JNK, PI3K/AKT and p38 signalling pathway mediated by DHK in RAW264.7 cells. (A) Cells were pre‐treated with inhibitors ERK (PD98059, 30 μM), PKC (GF109203X, 2.5 μM), JNK (SP600125, 25 μM), PI3K/AKT (LY294002, 30 μM) or MAPK p38 (SB203580, 20 μM) followed by DHK (12.5 μM) treatment for 1 h. The nuclear Nrf2 expression were evaluated by western blotting. (B) Treatment of cells with DHK (12.5 μM) for 0, 15, 30, 45 and 60 min respectively and Western blotting were performed for the proteins JNK, PI3K/AKT and MAPK p38.

The role of kinase pathways in Nrf2 phosphorylation and nuclear translocation in DHK‐treated RAW264.7 cells was identified. Cells were treated beforehand with PD98059 (ERK, 30 μM), GF109203X (PKC, 2.5 μM), SP600125 (JNK, 25 μM), LY294002 (PI3K/AKT, 30 μM) or SB203580 (MAPK p38, 20 μM) for 1 h. The results revealed that DHK (12.5 μM for 4 h)‐triggered nuclear Nrf2 expression was noticeably decreased in the SB203580‐, LY294002‐ and SB203580‐pretreated RAW264.7 cells, signifying that JNK, PI3K/AKT and p38 pathways regulated Nrf2 nuclear translocation in DHK‐treated RAW264.7 cells (Figure 7A). Additionally, the effects of DHK (12.5 μM for 0–60 min) on JNK, PI3K, AKT and p38 phosphorylation were examined. Western blotting data showed that DHK enhanced p‐JNK, p‐PI3K, p‐AKT and p‐p38 expression in RAW264.7 cells (Figure 7B).

### 3.14 DHK Suppressed mtROS‐Induced NLRP3 Inflammasome Activation and IL1β Expression via Mitophagy Induction in LPS/ATP‐Instigated RAW264.7 Cells

**FIGURE 8:**
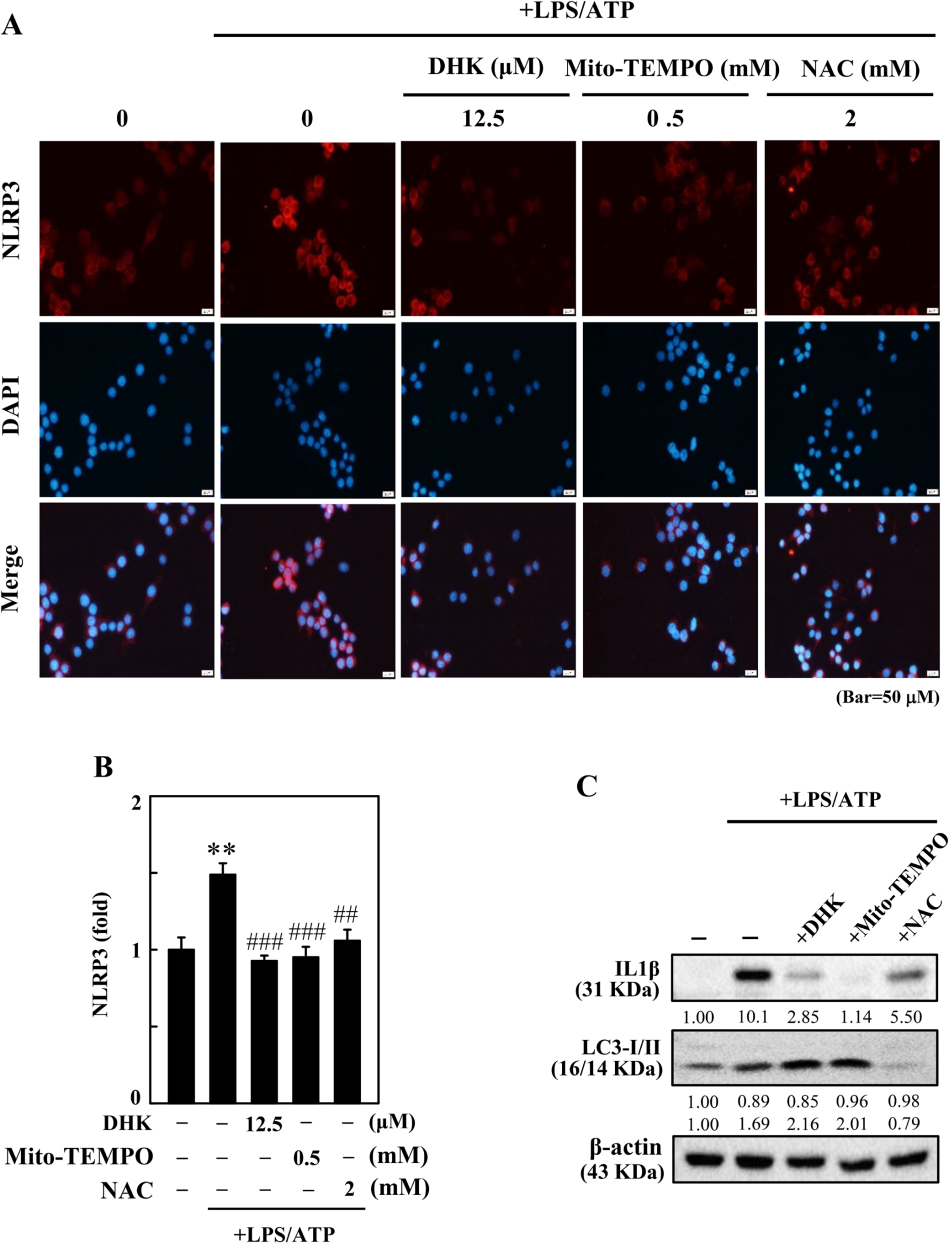
DHK inhibits ROS‐mediated NLRP3 inflammasome activation through autophagy induction and Nrf2 activation in LPS/ATP‐stimulated RAW264.7 macrophages. Cells were pre‐treated with DHK (12.5 μM), Mito‐TEMPO (0.5 mM) or NAC (2 mM) for 1 h, and then stimulated with LPS (1 μg/mL) for 5.5 h following by ATP (5 mM) for 0.5 h. (A, B) The nuclear localization of NLRP3 were visualised by immunofluorescence staining. (C) IL1β and LC3‐I/II expression was examined by Western blotting.

In response to LPS/ATP stimulation, mitochondria‐derived ROS (mtROS) from damaged mitochondria are linked to NLRP3 inflammasome‐activated IL1β expression [43]. Cells were incubated with DHK (12.5 μM), Mito‐TEMPO (0.5 mM, a mtROS inhibitor), and NAC (2 mM, a ROS inhibitor) for 1 h, and NLRP3 expression was monitored using immunofluorescence staining in LPS/ATP‐incited RAW264.7 cells. We found that DHK, Mito‐TEMPO and NAC treatment curbed NLRP3 expression in LPS/ATP‐instigated RAW264.7 cells (Figure 8A,B). Next, the Western blotting displayed that DHK, Mito‐TEMPO and NAC pretreatment attenuated IL1β expression in LPS/ATP‐induced RAW264.7 cells (Figure 8C). These findings implicated that inhibition of ROS including mtROS generation is involved in the DHK‐suppressed NLRP3 inflammasome and IL1β expression. During mitophagy, mitochondria are encapsulated into vesicles which are coated with autophagosomal LC3‐II. To ascertain the role of mitophagy, cells were pretreated with DHK, NAC or Mito‐TEMPO, and then modulations in the expression of LC3‐II were evaluated by Western blotting. Our findings showed that LC3‐II expression was prominently boosted by DHK and Mito‐TEMPO, but this outcome was inhibited under NAC pretreatment, in LPS/ATP‐stimulated RAW264.7 cells (Figure 8C). Together, the above findings unveiled that mitophagy plays a role in the DHK‐suppressed LPS/ATP‐stimulated mtROS‐triggered NLRP3 inflammasome and IL1β expression in RAW264.7 cells.

### 3.15 DHK Decreased Total Cells, Neutrophils, TNFα and IL1β Expression From BALF From LPS‐Stimulated BALB/c Mouse

**FIGURE 9:**
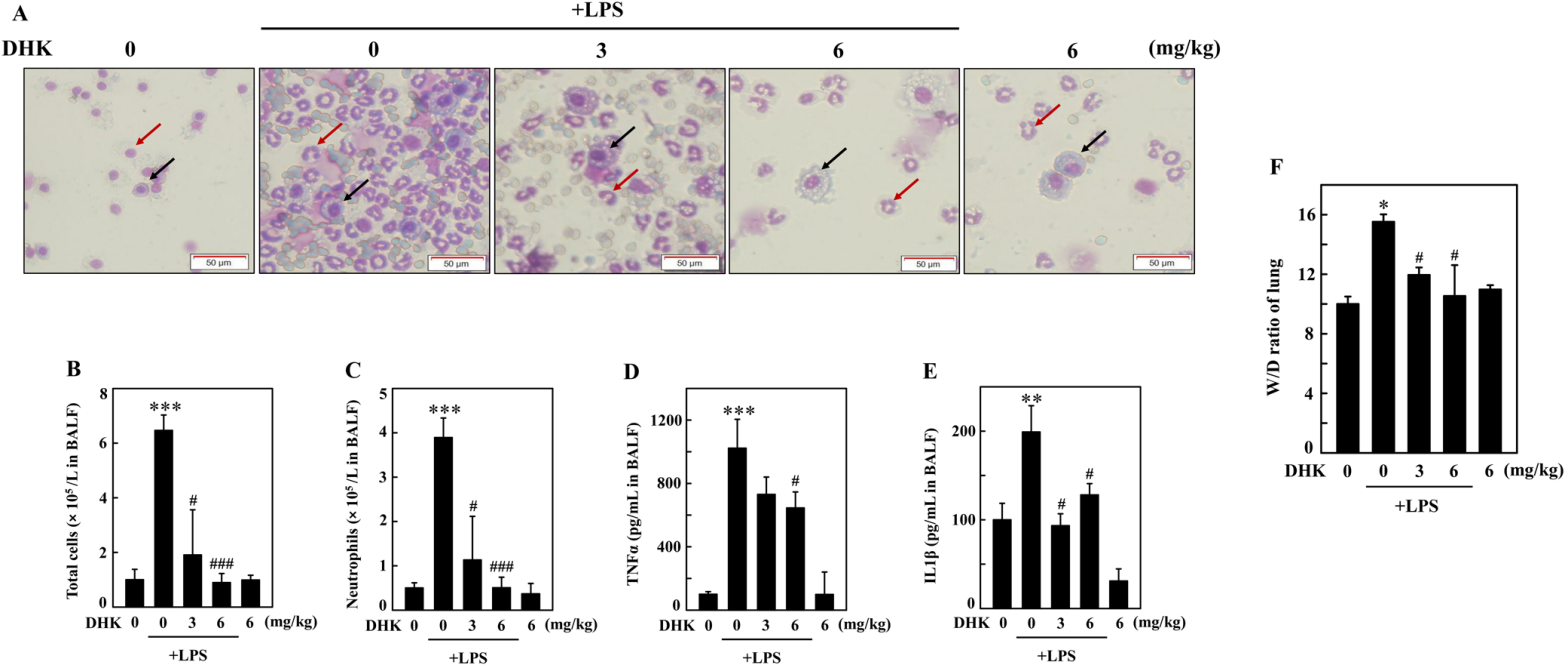
DHK decreased total cells, neutrophils, TNFα and IL1β expression from BALF from LPS‐stimulated BALB/c mouse. Mice (*n* = 6) were pretreated with DHK (0, 3, or 6 mg/kg) for 4 h and then LPS (1 mg/kg) was injected (intraperitoneal) for 24 h. After mice were anaesthetised, the bronchoalveolar lavage fluid (BALF) was collected. (A–C) BALF was examined using Wright‐Giemsa staining, and then total cells and neutrophils were counted under microscope. (D, E) TNFα and IL1β expression from BALF was assayed by ELISA. (F) After specified treatments, the mice were sacrificed, and the left lung were collected. The wet/dry weight ratio of the lung of LPS‐stimulated BALB/c mice were calculated as described in the methodology section.

To evaluate the effect of DHK on proinflammatory changes incurred upon LPS administration, mice were pretreated with DHK (0, 3 or 6 mg/kg for 4 h), and then (intraperitoneal) injected with LPS (1 mg/kg). After 24 h, BALB/c mice were anaesthetised, and the bronchoalveolar lavage fluid (BALF) was collected for further assessment. The activated neutrophils and macrophages during inflammation are known to produce higher levels of TNFα, IL1β, and other inflammatory mediators [44]. In the current study, Wright‐Giemsa staining revealed that total cells and neutrophils were markedly increased (Figure 9A–C), whereas the ELISA assay disclosed substantially amplified TNFα and IL1β expressions (Figure 9D,E) from BALF from LPS‐injected BALB/c mice. However, DHK administration significantly attenuated total cells, neutrophils, TNFα and IL1β to untreated control levels from BALF from LPS‐injected BALB/c mice.

### 3.16 DHK Decreased Wet/Dry Weight Ratio of Lung Tissue From LPS‐Stimulated BALB/c Mouse

The neutrophil intrusions and pulmonary oedema in the lung tissues are marked by the wet/dry weight ratio of lung tissue [45]. This study showed that the wet/dry weight ratio of (left) lung tissue was increased in LPS‐stimulated BALB/c mice; however, it was significantly decreased by DHK treatment (Figure 9F), disclosing that DHK decreased lung neutrophil intrusions and pulmonary edema in LPS‐injected BALB/c mice.

### 3.17 DHK Suppressed NLRP3 Inflammasome Activation and Triggered Antioxidant Nrf2 Signalling Pathway in LPS‐Stimulated BALB/c Mouse

**FIGURE 10:**
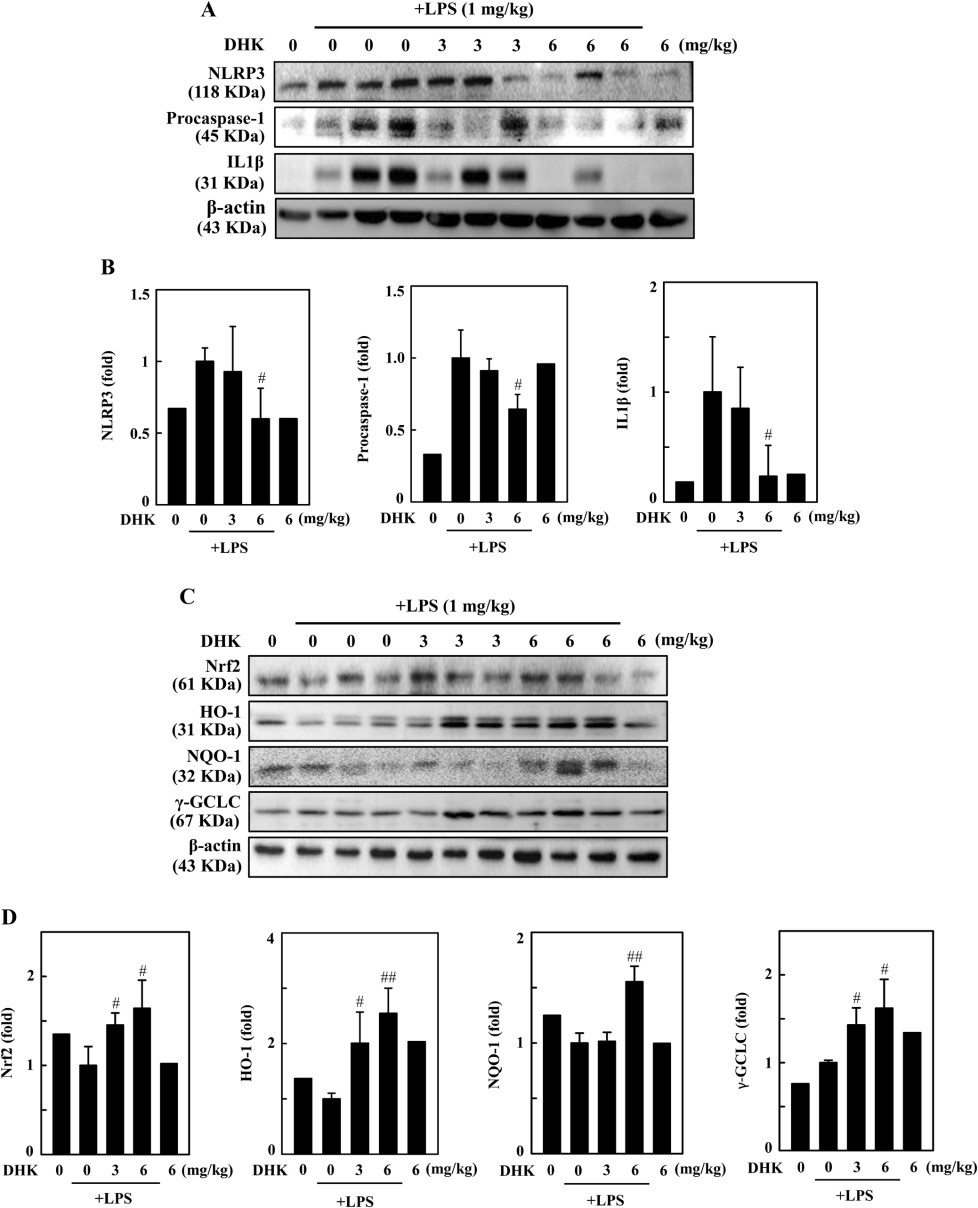
DHK activated Nrf2 signalling pathway and suppressed NLRP3 inflammasome activation in LPS‐stimulated BALB/c mouse. Mice (*n* = 6) were pretreated with DHK (0, 3 or 6 mg/kg) for 4 h followed by LPS (1 mg/kg, intraperitoneal) injection for 24 h. The mice were sacrificed, and the right lung tissues were collected for immunoblotting. (A, B) NLRP3, procaspase‐1, IL1β expression was examined by western blotting. (C, D) Nrf2, HO‐1, NQO‐1 and γ‐GCLC expression was examined by western blotting.

The consequence of DHK treatment on LPS‐stimulated inflammasome activation in BALB/c mice was investigated by pretreatment with DHK (0, 3 or 6 mg/kg) for 4 h and then subjected to LPS (1 mg/kg, intraperitoneal injection) for 24 h. The results showed that LPS‐stimulated BALB/c mice exhibited markedly enhanced protein expressions of IL1β, NLRP3 and procaspase‐1 as examined by Western blotting (Figure 10A,B). Treatment with DHK prominently reduced NLRP3 inflammasome markers (NLRP3, procaspase‐1 and IL1β) in the lung tissues of LPS‐injected BALB/c mice (Figure 10A,B). In addition, our data revealed that DHK triggered the antioxidant transcription factor Nrf2 and enzymes γ‐GCLC, HO‐1 and NQO‐1 protein expressions in LPS‐stimulated BALB/c mice, as assayed by Western blotting (Figure 10C,D). Treatment with DHK enhanced the Nrf2 signalling pathway (γ‐GCLC, HO‐1 and NQO‐1) in the lung tissues of LPS‐treated BALB/c mice (Figure 10C,D).

### 3.18 DHK Induced Mitophagy in LPS‐Stimulated BALB/c Mouse

**FIGURE 11:**
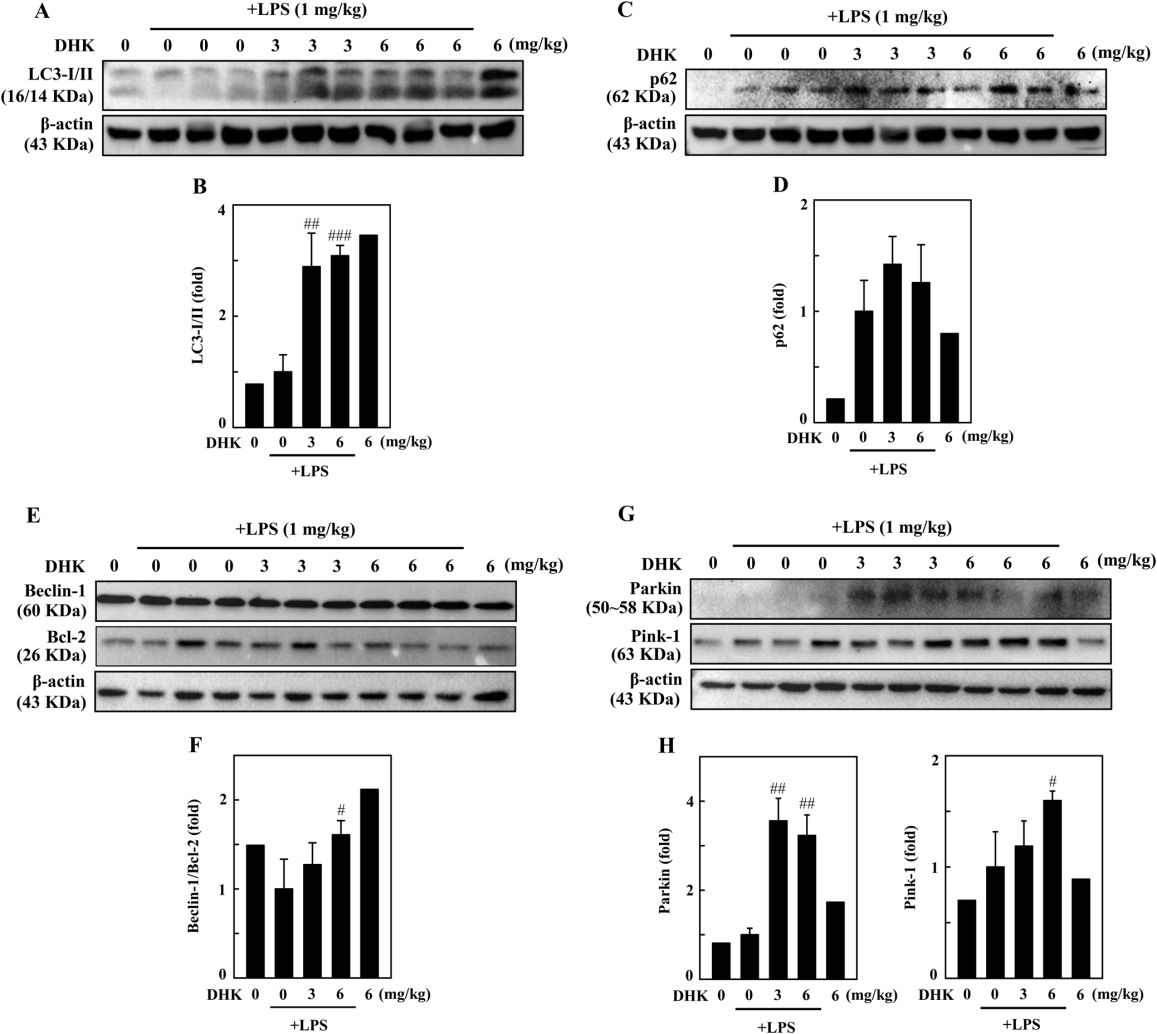
DHK provoked mitophagy in LPS‐stimulated BALB/c mouse. Mice (*n* = 6) were pretreated with DHK (0, 3 or 6 mg/kg) for 4 h followed by LPS (1 mg/kg, intraperitoneal) injection for 24 h. The mice were sacrificed, and the right lung tissues were collected for immunoblotting. (A–D) LC3‐I/II and p62 expression was examined by western blotting. (E, F) Beclin‐1 and Bcl‐2 expression was examined by western blotting. (G, H) Parkin and Pink‐1 expression was examined by western blotting.

To examine the effect of DHK on mitophagy induction, the BALB/c mice were treated beforehand with DHK (0, 3, or 6 mg/kg) for 4 h, followed by intraperitoneal injection with LPS (1 mg/kg). The Western blotting displayed that increasing autophagosome marker LC3‐I/II and p62 (Figure 11A–D), Beclin‐1/Bcl‐2 ratio (Figure 11E,F), and mitophagy specific markers Parkin and Pink‐1 (Figure 11G,H) were substantially enhanced under DHK treatment in the lung tissues of LPS‐injected BALB/c mice, suggesting that DHK provoked mitophagy in LPS‐stimulated BALB/c mice.

## 4 Discussion

**FIGURE 12:**
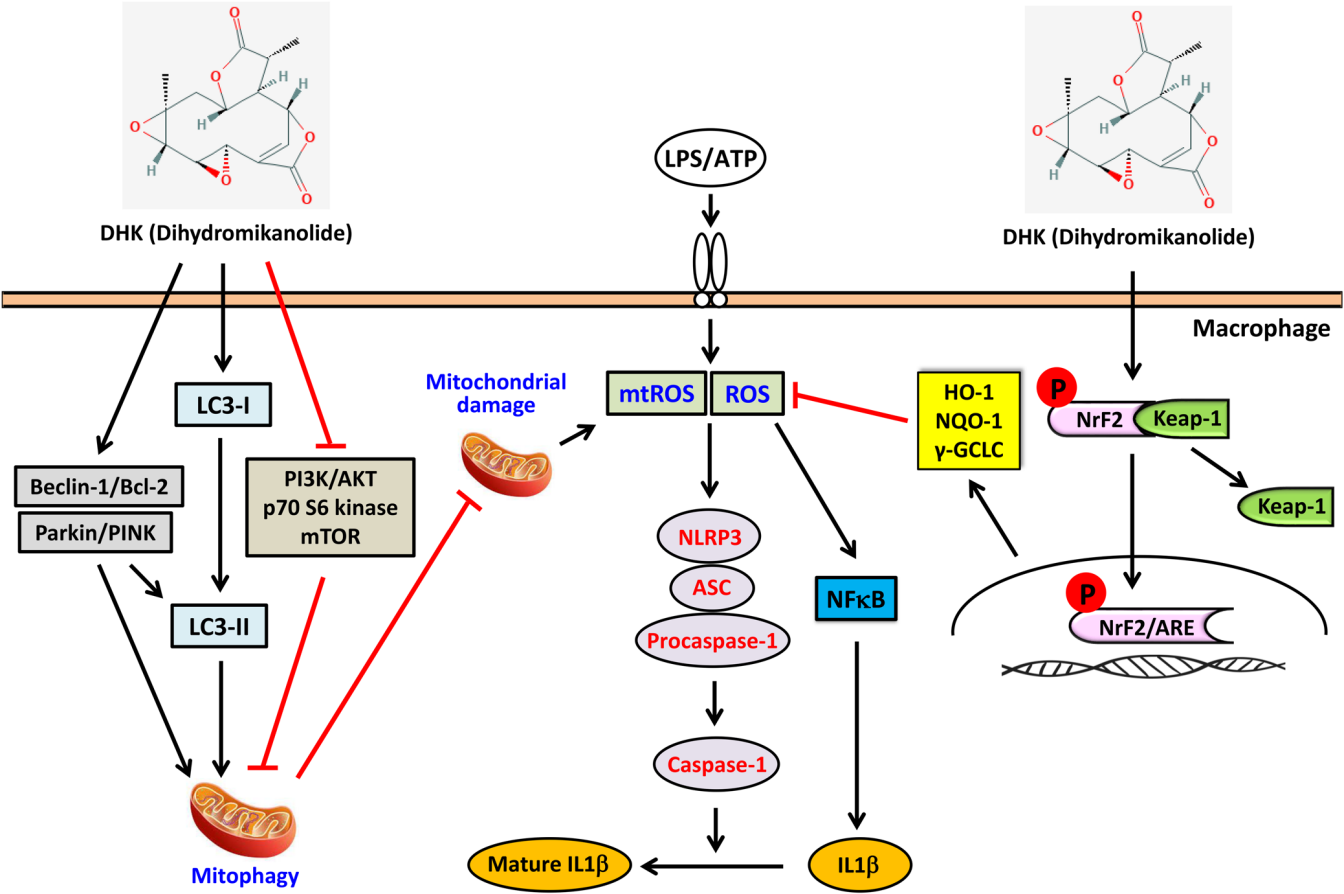
DHK inhibits ROS‐mediated NFκB and NLRP3 inflammasome activation through mitophagy induction and antioxidant Nrf2 activation in LPS/ATP‐stimulated macrophages and in vivo mice model.

*Mikania* extracts have been used widely in traditional medicine based on ethnobotany as an anti‐inflammatory, vermifuge, fever, anti‐rheumatic and analgesic agent. Dihydromikanolide (DHK) is a natural sesquiterpene lactone isolated from numerous *Mikania* species (such as *Mikania micrantha* and *Mikania cordata* ) [19, 21, 46]. In previous study, DHK was shown to inhibit pathological inflammation in TPA‐induced edema in the ear of mouse model [23]. To our knowledge, this is the foremost study that showed that DHK suppressed ROS‐triggered NFκB and NLRP3 inflammasome activation through the antioxidant Nrf2 pathway and mitophagy induction in LPS/ATP‐stimulated RAW264.7 macrophages (Figure 12).

LPS, a chief constituent of the exterior membrane of Gram‐negative bacteria, is a powerful activator of macrophages that causes the release of multiple pro‐inflammatory cytokines [47]. ATP is actively secreted in response to cellular stress and tissue damage in the extracellular environment [48]. Redox‐sensitive transcription factors, like NFκB, are then triggered within the LPS/ATP‐activated macrophages. Upon receiving the signal for NFκB nuclear translocation and transcriptional activation, the inhibitory protein IκB is phosphorylated and releases NFκB which then translocates into the nucleus for transcription of its target genes. The activated NFκB then induces their corresponding intermediary genes such as iNOS and COX‐2 as well as initiates various pro‐inflammatory molecules such as IL1β, which are primarily forcing the development and progression of inflammatory diseases [49, 50]. Our results disclosed that DHK significantly decreased LPS/ATP‐stimulated NFκB‐mediated iNOS and COX‐2 expression, which inferred the anti‐inflammatory outcome of DHK in RAW 264.7 cells.

The initiation of NLRP3 inflammasome is a precisely controlled mechanism that requires priming by ATP as well as an activation signal LPS [51]. One of the most characterised cytokines that is identified to be decisive in autoimmune disorders, pro‐inflammatory IL1β, is released with the activation of the inflammasome NLRP3, a multi‐protein complex that controls caspase‐1 activation [52, 53]. Caspase‐1, an interleukin‐1(IL1) converting enzyme, is a protease that cleaves the inactive precursor of IL1 to generate the mature IL1 form. The assembly of the NLRP3 complex together with autoproteolysis of procaspase‐1 produces active caspase‐1, which constitutes a hallmark of the human canonical inflammasomes [54, 55]. This study showed that LPS/ATP treatment enhanced NLRP3 inflammasome formation and procaspase‐1 expression; however, treatment with DHK repressed LPS/ATP‐induced NLRP3 activation, procaspase‐1 initiation, and then IL1β expression in RAW 264.7 macrophages. Our data concluded that DHK enhanced a negative regulatory loop against NLRP3 inflammasome in LPS/ATP‐incited RAW 264.7 cells. Previous studies have suggested that NLRP3 is typically positioned in the cytoplasm and exists in complexes with apoptosis‐associated speck‐like protein containing a CARD (ASC) and pro‐caspase‐1 in several immune cells, especially in macrophages, dendritic cells and T lymphocytes. The cytoplasmic localization of NLRP3 might promote inflammasome assembly, while nuclear enrichment might enhance the transcriptional function of the inflammasome [36, 37]. However, through Kpna2‐mediated nuclear translocation, NLRP3 negatively regulates lymphocyte Treg differentiation [56].

Previous research has demonstrated that the NLRP3 inflammasome is regulated by cell‐autonomous controlling feedback loops in which autophagy plays a prominent role [57]. The process of autophagy, a catabolic mechanism preserved evolutionarily, entails the production of vesicles called autophagosomes that capture damaged or nonfunctional macromolecules and cell organelles and combine with lysosomes (autolysosomes) to facilitate their degradation and clearance [58]. A stable connection develops between LC3‐II and the autophagosome membrane during autophagy. p62, also known as sequestosome 1 (SQSTM1), is a versatile ubiquitin‐binding protein that plays a key role in autophagy. p62 binds with ubiquitinated cargo and attaches to LC3 followed by autolysosomal degradation while undergoes self‐decomposition during autophagy [12, 57]. Our results revealed that DHK treatment augmented the LC3‐II accumulation and p62 expression was increased. Further, pre‐treatment of cells with early autophagy inhibitor 3‐MA (2.5 mM) significantly decreased LC3‐II expression suggested that DHK activated autophagy in LPS/ATP‐incited RAW264.7 cells.

The Bcl‐2 family of proteins functions as an important modulator of mitochondrial arbitrated apoptosis, acting as either an activator or inhibitor of apoptosis. Beclin‐1 and Bcl‐2 have a complex interplay, and Bcl‐2 can impede Beclin‐1's pro‐autophagic function [59]. Bcl‐2 expression was decreased; however, autophagic Beclin‐1 proteins increased considerably by DHK, as demonstrated by Western blotting results. The study showed that the ratio of Beclin‐1 to Bcl‐2 was augmented as DHK concentration increased, indicating that autophagy was enhanced in RAW 264.7 cells. The PI3K/AKT/mTOR pathways have a negative correlation with autophagy, which is crucial for inflammation and several chronic illnesses. The PI3K/AKT pathway activates downstream mTOR, which in turn phosphorylates the p70S6 kinase [60]. Our data showed that DHK significantly reduced PI3K, AKT, mTOR and p70S6 kinase expressions inferring that DHK‐activated autophagy plays a pivotal role in RAW 264.7 cells. Our results revealed that DHK inhibited LPS/ATP‐stimulated IL1β expression in RAW264.7 cells. Additionally, 3‐MA antagonised the actions of DHK in suppressing IL1β expression. Further, suppression of autophagy through LC3 silencing reversed DHK‐inhibited IL1β expression, confirming that DHK‐activated autophagy plays a critical role in LPS/ATP‐incited inflammation in RAW264.7 cells.

Nrf2 is an indispensable factor governing the expression of cytoprotective genes against LPS/ATP‐incited inflammation [42]. There exists a modulatory linkage between Nrf2 activation and the NFκB and NLRP3 inflammation [42, 49]. The current study exhibited that the anti‐inflammatory effects of DHK involved the initiation of antioxidant enzymes, γ‐GCLC, HO‐1 and NQO‐1 through the Nrf2 signalling cascade. HO‐1 is known to break down heme to produce biliverdin that is subsequently converted to bilirubin, which exerts antioxidant and anti‐inflammatory effects [61]. NQO‐1 helps in regenerating α‐tocopherol (vitamin E) which acts as an endogenous antioxidant after free radical attack [62]. γ‐GCLC is the first‐rate limiting enzyme of antioxidant glutathione synthesis [63]. Thus, treatment with the antioxidant Nrf2 activator DHK could be an amenable method to ameliorate the advancement of ROS‐mediated inflammatory diseases. In addition, autophagic p62/SQSTM1 has been linked at the interface between autophagy and the antioxidant Nrf2 pathway [64]. p62/SQSTM1 degraded Keap‐1 and then stimulated Nrf2 nuclear translocation; therefore, it increased the antioxidant HO‐1, NQO‐1 and γ‐GCLC expressions [64]. After Nrf2 knockdown, DHK‐inhibited IL1β expression in LPS/ATP‐incited RAW264.7 macrophages was significantly reversed. Together, the above data suggested that DHK repressed ROS‐mediated NFκB/NLRP3 inflammasome instigation and IL1β expression via antioxidant Nrf2 activation.

Phosphorylated Nrf2 activation and nuclear translocation have been shown to be regulated by several kinases [65, 66]. Previous studies have shown that phosphorylation of Nrf2 at serine/threonine residues by various kinases releases Keap‐1 from Nrf2, which is translocated to the nucleus to transcribe the antioxidant genes γ‐GCLC, HO‐1 and NQO‐1. While suppression of these kinases by specific inhibitors diminished nuclear Nrf2 translocation [65, 66]. Our data revealed that JNK, PI3K/AKT and p38 inhibitors remarkably suppressed the DHK‐induced nuclear Nrf2 translocation in RAW264.7 cells. The Nrf2 activation upon DHK treatment was consistent with DHK‐induced phosphorylated JNK, PI3K/AKT and p38 pathways. These results established that DHK‐triggered Nrf2 activation functioned via JNK, PI3K/AKT, and p38 pathways in RAW264.7 macrophages.

Damaged mitochondria in cells are removed through a specific type of autophagy known as mitophagy [41]. The Pink‐1 kinase and E3 ubiquitin ligase Parkin are recruited onto the depolarised mitochondrial outer membrane, which marks the mitochondria for autophagy‐mediated elimination [67]. Accretion of Parkin and Pink‐1 occurs in dysfunctional mitochondria to enable their segregation from the mitochondrial network and target these organelles for autophagic degradation [68]. Our data disclosed that DHK treatment enhanced Parkin and Pink‐1 expression, confirming that DHK induced mitophagy in RAW 264.7 cells. Importantly, there were increased autophagy markers including LC3‐II, p62/SQSTM1, Beclin‐1/Bcl‐2 ratio and decreased phosphorylated mTOR by DHK treatment in RAW 264.7 cells. Our results confirmed that a combinatorial effect of mitophagy and autophagy by DHK treatment antagonised the NFκB/NLRP3 inflammasome in RAW 264.7 macrophage cells.

Dysfunctional mitochondria upon LPS/ATP stimulation generate excessive mtROS leading to NFκB activation and NLRP3 inflammasome activation [69]. The removal of excessive mtROS generating dysfunctional mitochondria through mitophagy induction has been suggested as a probable way of inhibiting harmful levels of NLRP3 inflammasome activation [2]. Further, a mitochondria‐targeted ROS quencher, Mito‐TEMPO, suppresses IL1β secretion after ATP exposure [70]. Enhancing mitophagy inhibits an accretion of damaged mitochondria producing unnecessary mtROS, which suppresses the NLRP3 and IL1β expression [14]. This study showed that treatment with DHK, Mito‐TEMPO (a mtROS inhibitor), and NAC (a ROS inhibitor) significantly suppressed the LPS/ATP‐stimulated NLRP3 and IL1β expression in RAW264.7 macrophages. This suggested that ROS signalling cascades were involved in DHK‐inhibited NLRP3 inflammasome and IL1β expression. However, treatment with DHK or Mito‐TEMPO, but not NAC, in LPS/ATP‐stimulated RAW264.7 macrophages significantly increased LC3‐II accretion indicative of the occurrence of mitophagy. Our data concluded that DHK suppressed mtROS‐mediated NLRP3 inflammasome and IL1β expression via mitophagy induction in LPS/ATP‐incited RAW264.7 cells.

LPS stimulated inflammatory phenomenon has been extensively investigated in both in vitro and in vivo models encompassing physiology, immunology and metabolism [71, 72]. Similar studies conducted using traditional Chinese medicine ameliorated the acute lung injury in LPS administered mice [73]. This study also investigated the therapeutic potential of DHK in LPS administered BALB/c mice in vivo. We found that DHK decreased total cells and neutrophils, TNFα and IL1β expression in BALF in LPS stimulated BALB/c mice. DHK decreased wet/dry weight ratio of lung tissue in LPS stimulated BALB/c mice representing decrement of lung neutrophil intrusions and pulmonary oedema. Further, DHK alleviated LPS induced pathological alterations of lung through inhibiting NLRP3 inflammation (NLRP3, procaspase‐1, and IL1β), enhancing antioxidant Nrf2 activation (γ‐GCLC, HO‐1 and NQO‐1), and inducing mitophagy (LC3‐I/II, p62, Beclin‐1/Bcl‐2 ratio, Parkin and Pink‐1) in LPS stimulated BALB/c mice.

## 5 Conclusion

In summary, this is the first in vivo report to reveal the anti‐inflammatory activities of DHK through repression of the NFκB and NLRP3 inflammasomes through the Nrf2 pathway and mitophagy induction in mice against LPS challenge. Altogether, we found that DHK inhibited LPS/ATP‐triggered ROS generation, NFκB activation and iNOS and COX‐2 expression in RAW264.7 cells. DHK repressed ROS‐mediated NFκB and NLRP3 inflammasome activation as well as IL1β expression through Nrf2 activation. Notably, DHK attenuated mtROS‐arbitrated NLRP3 inflammasome and IL1β expression via mitophagy induction in LPS/ATP‐stimulated RAW264.7 cells. Further, the in vivo anti‐inflammatory properties of DHK were mediated through inhibition of the NFκB and NLRP3 inflammasomes via the Nrf2 pathway and mitophagy induction in LPS‐administered mice. As per our understanding, this is the primary study to report the anti‐inflammatory properties of DHK in both LPS/ATP‐stimulated RAW264.7 macrophages in vitro and LPS‐administered mice in vivo. Therefore, Dihydromikanolide may be a potential therapeutic agent for inflammatory diseases.

## Author Contributions

**You‐Cheng Hseu:** conceptualization (lead), funding acquisition (equal), investigation (lead), resources (supporting), writing – original draft (supporting), writing – review and editing (supporting). **Yu‐Fang Tseng:** investigation (supporting). **Jhih Ke‐Hseu:** conceptualization (equal), investigation (equal), methodology (equal), writing – review and editing (supporting). **Sudhir Pandey:** formal analysis (supporting), writing – original draft (lead), writing – review and editing (lead). **Siang‐Jyun Chen:** methodology (supporting), validation (supporting). **Kai‐Yuan Lin:** validation (supporting), visualization (supporting). **Hsueh‐Wei Chang:** validation (supporting), visualization (supporting). **Tzong‐Der Way:** validation (supporting), visualization (supporting). **Jhih‐Hsuan Hseu:** conceptualization (supporting), methodology (lead), resources (supporting), software (supporting), supervision (supporting), visualization (supporting), writing – review and editing (supporting). **Hsin‐Ling Yang:** conceptualization (equal), data curation (lead), formal analysis (supporting), funding acquisition (equal), methodology (lead), project administration (lead), resources (lead), software (lead), supervision (lead), validation (lead), writing – review and editing (supporting).**Chuan‐Chen Lee** (CCL): formal analysis (supporting).

## Conflicts of Interest

The authors declare no conflicts of interest.

## Data Availability

The data used in this study are included within the manuscript and would be available from the corresponding author on reasonable request.
